# Angiotensin-(1–7) Peptide Hormone Reduces Inflammation and Pathogen Burden during *Mycoplasma pneumoniae* Infection in Mice

**DOI:** 10.3390/pharmaceutics13101614

**Published:** 2021-10-04

**Authors:** Katie L. Collins, Usir S. Younis, Sasipa Tanyaratsrisakul, Robin Polt, Meredith Hay, Heidi M. Mansour, Julie G. Ledford

**Affiliations:** 1 Department of Immunobiology, College of Medicine, The University of Arizona, Tucson, AZ 85724, USA; collins.katieleanne@gmail.com; 2 Asthma and Airway Disease Research Center, Tucson, AZ 85724, USA; usirsyounis@gmail.com (U.S.Y.); sasipat@email.arizona.edu (S.T.); 3 Departments of Chemistry and Biochemistry, College of Science, The University of Arizona, Tucson, AZ 85721, USA; polt@u.arizona.edu; 4 Department of Physiology, College of Medicine, The University of Arizona, Tucson, AZ 85724, USA; mhay@email.arizona.edu; 5 BIO5 Institute, The University of Arizona, Tucson, AZ 85719, USA; mansour@pharmacy.arizona.edu; 6 Department of Medicine, Division of Translational & Regenerative Medicine, College of Medicine, The University of Arizona, Tucson, AZ 85724, USA; 7 Departments of Pharmacology/Toxicology and Pharmaceutical Sciences, College of Pharmacy, The University of Arizona, Tucson, AZ 85724, USA; 8 Department of Cellular and Molecular Medicine, College of Medicine, The University of Arizona, Tucson, AZ 85721, USA

**Keywords:** angiotensin-(1–7), *Mycoplasma pneumoniae*, asthma, inflammation, macrophages

## Abstract

The peptide hormone, angiotensin (Ang-(1–7)), produces anti-inflammatory and protective effects by inhibiting production and expression of many cytokines and adhesion molecules that are associated with a cytokine storm. While Ang-(1–7) has been shown to reduce inflammation and airway hyperreactivity in models of asthma, little is known about the effects of Ang-(1–7) during live respiratory infections. Our studies were developed to test if Ang-(1–7) is protective in the lung against overzealous immune responses during an infection with *Mycoplasma pneumonia* (Mp), a common respiratory pathogen known to provoke exacerbations in asthma and COPD patients. Wild type mice were treated with infectious Mp and a subset of was given either Ang-(1–7) or peptide-free vehicle via oropharyngeal delivery within 2 h of infection. Markers of inflammation in the lung were assessed within 24 h for each set of animals. During Mycoplasma infection, one high dose of Ang-(1–7) delivered to the lungs reduced neutrophilia and *Muc5ac*, as well as *Tnf-α* and chemokines (*Cxcl1*) associated with acute respiratory distress syndrome (ARDS). Despite decreased inflammation, Ang-(1-7)-treated mice also had significantly lower Mp burden in their lung tissue, indicating decreased airway colonization. Ang-(1–7) also had an impact on RAW 264.7 cells, a commonly used macrophage cell line, by dose-dependently inhibiting TNF-α production while promoting Mp killing. These new findings provide additional support to the protective role(s) of Ang1-7 in controlling inflammation, which we found to be highly protective against live Mp-induced lung inflammation.

## 1. Introduction

Angiotensin-(1–7) or Angiotensin_1–7_, here forth referred to as Ang-(1–7), is an endogenous peptide that is produced from Angiotensin II by angiotensin-converting enzyme 2 (ACE2). Ang-(1–7) has been shown to inhibit inflammatory actions of Angiotensin II through interactions with the Mas receptor. This axis is known to counteract and balance the vasoconstrictive effects of the renin-angiotensin system [1]. Recent studies have described how Ang-(1–7) opposes this inflammatory pathway, and as such, long-term administration of Ang-(1–7) has been shown to reduce oxidative stress and pro-inflammatory cytokine production in animal models [1]. Additional clinical positive effects on Ang-(1–7) reported include prevention of cardiac output reduction, cardiomyocyte size normalization, interstitial fibrosis and lung fibrosis reduction, decreased collagen deposition, and prevention of oxidative damage in vessels [2,3,4,5]. Papinska et al. proposed that Ang-(1–7) treatment inhibits all of the contributing factors that lead to structural remodeling in the lungs [1]. Other efforts have also delved into mechanistic pathways by which the ACE2/Ang-(1-7)/Mas axis acts to inhibit certain inflammatory pathways, one of which is thought to be directly involved in suppressing ERK and NF-κB-dependent signaling pathways [6,7]. 

A key regulator of heart, vascular, kidney, and brain function is the ACE2 enzyme, which is an important component of the well-described renin–angiotensin system (RAS) [8]. The ACE-AngII-AT1R system is thought to be opposed physiologically, and balanced by the ACE2–Ang-(1-7)-Mas system [9,10,11]. These two separate enzymatic pathways of RAS are thought to be involved in functionally balancing reactive oxygen species (ROS), nitric oxide (NO) production and inflammation, in several areas of the body, including peripheral tissues and the brain [8,9,12]. The beneficial and tissue protective effects of Ang-(1–7) are generated by Ang-(1–7) binding to the G-protein-coupled Mas receptor. Studies in our labs have shown that Ang-(1–7) and its derivatives decrease IL-6, IL-7, TNF-α, and macrophage influx into the brain [13], and reversed cognitive impairment caused by systemic inflammatory disease caused by heart failure. Studies by others have shown activation of the ACE2–Ang-(1-7)-Mas system is associated with inhibition of the ERK1/2 pathway [14] and inhibition of leukocyte influx and inflammatory tissue damage.

Mas receptors are present in the lung epithelium, and with regard to resolving pulmonary inflammation, connections between Ang-(1–7) and the Mas receptor have been observed [15]. Research is ongoing to better understand the mechanisms of the ACE2/Ang-(1-7)/Mas axis and the role these cellular and molecular actions play within acute lung injury and lung fibrogenesis [16]. Along these lines, studies have also demonstrated the importance of the Mas receptor-dependent anti-inflammatory effects of Ang-(1–7) in the OVA model of allergic airways disease [6,17,18]. A considerable number of in vivo studies demonstrates the beneficial actions of the ACE2/ANG-(1–7) axis in acute lung injury in several animal models [19,20,21].

Outside of the respiratory system, Ang-(1–7) has also shown function as a vasodilator with anti-thrombotic and anti-proliferative effects in pre-clinical trials [3,22]. These biological characteristics have suggested potential indications of Ang-(1–7) as a therapeutic drug option for cardiovascular disease conditions, such as hypertension, as well as a prospective cancer therapy [23,24]. Formulations of Ang-(1–7) have been administered in several clinical trials for the treatment of a variety of conditions, including pre-eclampsia, as well as for ovarian cancer patients undergoing chemotherapy [25,26]. We have previously published results showing cognitive protection following treatment with the native and glycosylated-Ang-(1-7), in our model of inflammation-induced cognitive impairment [13,27]. 

Additional studies from our group have shown that Ang-(1–7) derivatives decrease TNF-α, IL-7, IL-6, MCP1, and G-CSF while increasing the protective cytokine, IL-10, and inhibiting macrophage influx into the brain [13]. Cytokine release syndrome (CRS) or “cytokine storm” is defined as a massive systemic inflammatory response that can be triggered by a variety of factors including viruses, influenza, bacterial infections, and certain antibody-based therapeutic drugs, most common among these being chimeric antigen receptor (CAR)-T cell infusion in hematological patients [28]. The trigger stimulus typically results in the lysis of immune cells and release of IFN−γ and TNF−α. This in turn results in a feed-forward cascade of the release of pro-inflammatory cytokines from macrophages and endothelial cells leading to a cytokine storm [28]. In the CAR-T cell associated CRS patients, IL-6 is thought to be the driver in clinical symptoms with tocilizumab, a monoclonal antibody directed against IL-6 receptors, as a first line of therapy [29,30]. Given that Ang-(1–7) and derivatives are pluripotent and known to inhibit many of the cytokines activated in the “cytokine storm”, our study was set up to test the efficacy of Ang-(1–7) in controlling inflammation during live pulmonary infection with Mp.

Infection with the Mp, also known as “walking pneumonia”, is a common cause of respiratory illnesses in children and often leads to exacerbations in asthma and COPD patients [31,32,33,34]. Colonization of Mp in the airway is a potent inducer of TNF-α production and is associated with increased neutrophilia in the lung [31]. The objective of the present study was to determine if treatment with Ang-(1–7) peptide would provide protective and anti-inflammatory effects in Mp-challenged mice. We discovered that when given within 2 h of Mp infection, one dose of Ang-(1–7) delivered to the lungs significantly reduced neutrophilia and *Muc5AC*, as well as TNF-α and KC. Despite decreased inflammation, Ang-(1–7) treatment also resulted in significantly lower Mp burden in the lung tissue, which in vitro studies suggest may be through Ang-(1-7)-dependent enhanced macrophage killing mechanisms. To the authors’ knowledge, this study is the first to report these findings.

## 2. Methods

### 2.1. Animal Models

BALB/c male mice (~22–27 grams) were purchased from Jackson labs (Bar Harbor, ME, USA) for use in this model of Mp infection. Each experimental group was approximately 6–8 weeks of age and included of a vehicle group, a Mp infection only group, a Mp infection given a low dose Ang-(1–7) (Sigma, St. Louis, MO, USA) treatment group, and a Mp infection given high dose Ang-(1–7) treatment group. All animals were sacrificed after 24 h of infection for analysis of inflammation. The study was repeated twice with an n = 10 for each experimental condition. All studies were on protocols approved and in accordance with the University of Arizona Institutional Animal Care and Use Committee (IACUC) on protocol number 15-575, with approval dates 2/06/2018 and 2/06/2021.

### 2.2. Pathogen-Challenge and Drug Treatment

Mp was given via intranasal instillation at 1 × 10^8^ Mp delivered in 50 μL per mouse, as previously described [35]. The experimental vehicle group, which did not receive the Mp, received a dose of sterile saline via intranasal instillation. Ang-(1–7) was given two hours post-infection via forced oropharyngeal instillation at a low dose of 0.3 mg/kg and a high dose of 1.0 mg/kg, while mice were under isoflurane anesthesia. Doses of Ang-(1–7) were based on previous publications in which Ang-(1–7) was active in repressing inflammation in murine models in the 0.3 and 1.0 mg/kg range [6,13]. Forced oropharyngeal delivery is used in place of traditional intratracheal incisions as we have previously shown [36]. All members performing this procedure have been trained with Evan’s blue dye to insure their technique results in lung delivery, and not delivery to the gut. The animals that were not treated with Ang-(1–7) received sterile saline via oropharyngeal instillation. Bronchoalveolar lavage (BAL) fluid and lungs from the mice were harvested twenty-four hours post-infection for cellular analysis, qPCR, ELISA, and histological analysis of inflammatory biomarkers.

### 2.3. Bronchoalveolar Lavage (BAL) Fluid Cell Counts and Cell Differentiation

Twenty-four hours post-infection, mice were euthanized through intraperitoneal injection of a lethal dose of urethane. Lungs were gently flushed with 1.25 mL of PBS (0.1 mM EDTA) to obtain bronchoalveolar lavage fluid (BALF) via a cannulated trachea. The cell-free lavage fluid was used to assess cytokines and chemokines. BALF cells were enumerated by an automated cell counter (Countess, Thermo Fisher Scientific, Waltham, MA, USA) with Trypan blue exclusion for cell viability. Differential cell counts were assessed by use of the Easy III Stain Kit.

### 2.4. Lung Tissue Preparation for Histological Analysis

Both left and right lung lobes were collected from each mouse subject following BALF collection. Right lung lobes were collected and processed for RNA extraction. Left lung lobes were preserved by immersion in 10% formalin. Left lung lobes were transferred to immersion in 70% ethanol at least 3 days following collection of samples. Left lung samples were processed for hematoxylin and eosin (H&E) staining, and sections of each lung sample were scored in a blinded manner according to a standard scale: 0 = only alveolar macrophages were detected throughout entire tissue section; 1 = very few neutrophils observed throughout entire tissue section; 2 = neutrophils present in alveolar airspaces only; 3 = neutrophils observed in alveolar airspaces and in lymphatics; 4 = neutrophils observed in alveolar airspaces, in lymphatics and in the lumen of large airways.

### 2.5. RT-PCR Analysis

Right lung lobe tissue was collected from each subject and processed for RNA extraction, cDNA synthesis, and real-time polymerase chain reaction (RT-PCR) analysis. Bio-Rad^TM^ (Bio-Rad laboratories, Hercules, CA, USA) cDNA Synthesis kit was used to synthesize DNA from 1 μg of total RNA. Quanta bio PerfeCTa SYBR Green Supermix (Quanta BioSciences, Gaithersburg, MD, USA) was used to perform RT-PCR. The gene expression of Mp P1-adhesin, TNF-α, Muc5ac, and KC were measured in mice lung tissue by RT-PCR. The mammalian housekeeper gene cyclophilin was used for expression level normalization.

### 2.6. Growth and Stimulation of RAW 264.7 Cells

RAW 264.7 cells were purchased from ATCC (TIB-71^™^, Manassas, VA, USA) and grown according to standard conditions. For TNF-α stimulation, RAW cells were grown until confluent in 24-well culture plates (~500,000 cell per well). On the day of challenge, media was removed and replaced with media containing differing doses of Ang-(1–7) or vehicle control, 30 min prior to Mp stimulation. Mp was added at a MOI of 10:1 and allowed to stimulate cells for 4 h, after which the media was removed and TNF-α levels determined by ELISA. For Mp killing assays, RAW cells were seeded at a density of ~100,000 cells per well into a 96-well tissue culture plate and allowed to adhere (~4 h). Media was removed and prepared Mp was added at a MOI of 2:1 (200,000 Mp to 100,000 seeded cells) with increasing doses of Ang-(1–7) or vehicle control (saline). After 18 h, a 10-μL sample was taken from each well, diluted 1:100 in SP4 media and plated on PPLO agar plates. After 2 weeks of incubation at 35 °C with no CO_2_, Mp counts were enumerated with the aid of a microscope at 4x magnification. At the dosing range used for these studies, Ang-(1–7) did not appear to directly impact Mp growth alone as no differences in colony counts were detected when Ang-(1–7) was added to Mp alone in the range of 0–0.5 μg/mL. Cell viability was assessed over a 24-h period, in which increasing doses of Ang-(1–7) were added by Trypan blue exclusion with the aid of an automated cell counter (Countess, Thermo Fisher Scientific, Waltham, MA, USA). Doses were examined in triplicate wells.

### 2.7. ELISA Analysis

Media from the RAW cell studies (diluted 1:10–1:100) were examined for TNF-α protein levels according to standard methods of the Mouse TNF-α ELISA MAX Deluxe Set protocol (R&D Biosystems, Minneapolis, MN, USA).

### 2.8. Statistical Analysis

Prism software (GraphPad, version 8.1.2) was used for all statistical analyses. Data comparisons and analysis for significance was performed using ordinary one-way ANOVA with Turkey’s multiple comparisons test. Reported significance if * *p* < 0.05, ** *p* < 0.01, *** *p* < 0.001, and **** *p* < 0.0001.

## 3. Results

### 3.1. Effect of Ang-(1–7) on Inflammatory Cells in the BALF Post Mp-Infection

**Figure 1:**
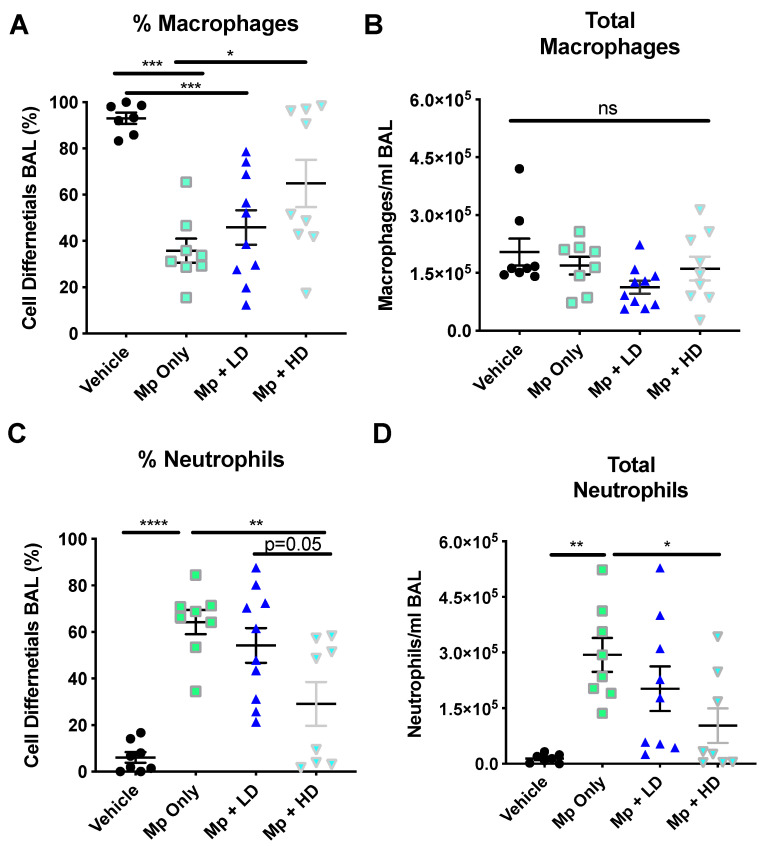
The effect of Ang-(1–7) on inflammatory cell recruitment in *Mycoplasma pneumoniae*–challenged BALB/c mice. A–D) Mice were infected with Mp (or vehicle control) and 2 h later were treated with Ang-(1–7) at high (1 mg/kg body weight) or low (0.3 mg/kg body weight) doses. Total cells present in the BALF were assessed by differential staining. (**A**) The percentage, (**B**) total macrophages, (**C**) the percentage, and (**D**) total neutrophils present in the BAL 24 h post infection are reported. Data are expressed in mean ± SEM. n = 8–10 mice/group, two independent experiments combined. * *p* < 0.05, ** *p* < 0.01, *** *p* < 0.001, **** *p* < 0.0001 by one-way ANOVA for multiple comparisons.

Infection with *Mycoplasma pneumoniae* resulted in a significant decrease in the percentage of macrophages, while increasing the percentage of neutrophils present in the BALF as compared to non-infected controls (Figure 1A,C). The total number of macrophages remained consistent (Figure 1B); however, the total number of neutrophils in the Mp-infected only group was significantly increased compared to non-infected vehicle control (Figure 1D). The group receiving the high dose (1.0 mg/kg) Ang-(1–7) treatment following Mp infection had significantly fewer neutrophils, as measured as a percentage of cells recovered and by total neutrophil counts as compared to the Mp infection only group (Figure 1C,D). The group receiving a low dose (0.3 mg/kg) Ang-(1–7) treatment had a significant decrease in the total number of neutrophils as compared to the group with Mp infection only (Figure 1D).

### 3.2. Effect of Ang-(1–7) on Mp Burden in Lung Tissue

**Figure 2:**
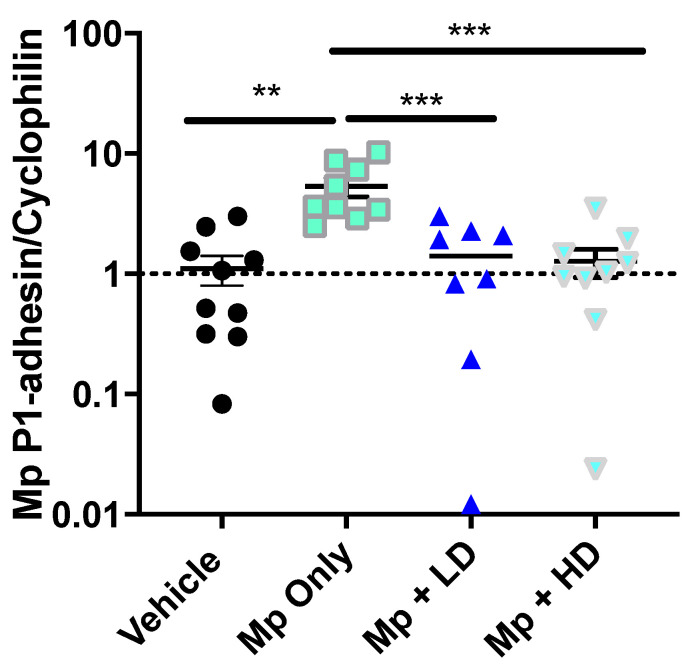
The effect of Ang-(1–7) on *Mycoplasma pneumoniae* burden in lung tissue. Mp burden was determined in lung tissue by RT-PCR for Mp-specific *P1-adhesin* gene expression relative to housekeeper, *Cyclophilin*. Data are expressed in mean ± SEM. n = 8–10 mice/group, two independent experiments combined. ** *p* < 0.01, *** *p* < 0.001 by one-way ANOVA for multiple comparisons.

Colonization and infection by Mp occurs through adhesion of the bacteria to cells in the host respiratory tract [37]. Adhesin-P1, an Mp specific adhesin protein, was used to quantify the Mp burden from lung samples of the infected mice [35]. The expression of adhesin-P1 was measured by PCR. As shown in Figure 2, there was a significant decrease in Mp burden among both the low and high dose Ang-(1–7) treatment groups when compared to Mp infected only mice.

### 3.3. Effect of Ang-(1–7) on Muc5ac, Cytokines, and Chemokines during Mp Infection

**Figure 3:**
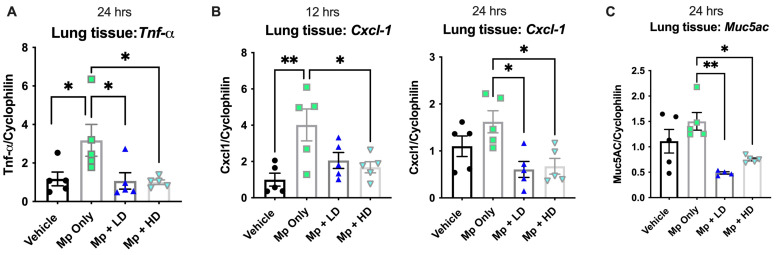
The effect of Ang-(1–7) on indices of inflammation during infection with *Mycoplasma pneumoniae*. (**A**) *Tnf-α, (***B**) *Cxcl1* (KC), and (**C**) *Muc5AC* gene expression were examined in lung tissue by RT-PCR in a subset of mice at either 12 h or 24 h post infection, as indicated. Data are expressed in mean ± SEM, relative to housekeeper gene expression. n = 5 mice/group and representative of two independent experiments. * *p* < 0.05, ** *p* < 0.01 by one-way ANOVA.

Typical markers associated with Mp infection were assessed to determine if Ang-(1–7) would have an impact on inflammation: the mucin gene *Muc5AC*, the cytokine TNF-α, and the neutrophil recruiting KC (*Cxcl1)*. Both low dose and high dose Ang-(1–7) treatments led to significantly decreased *Tnf-α* and *Cxcl1* gene expression in lung tissue following Mp infection (Figure 3A,B). *Cxcl1* levels were elevated 12 h after Mp infection, which were significantly decreased by the high dose of Ang-(1-7). While *Cxcl1* levels were lower by 24 h of infection compared to 12 h of infection, Ang-(1–7) treated groups continued to have reduced expression at both the low and high doses. Mp infection led to a slight increase in *Muc5AC* by 24 h; however, both Mp-infected groups treated with either low or high doses of Ang-(1–7) had significantly lower *Muc5ac* gene expression in lung tissue as compared to Mp-infected (Figure 3C). 

### 3.4. Effect of Ang-(1–7) Treatment on Lung Tissue Inflammation in Histological Sections

**Figure 4:**
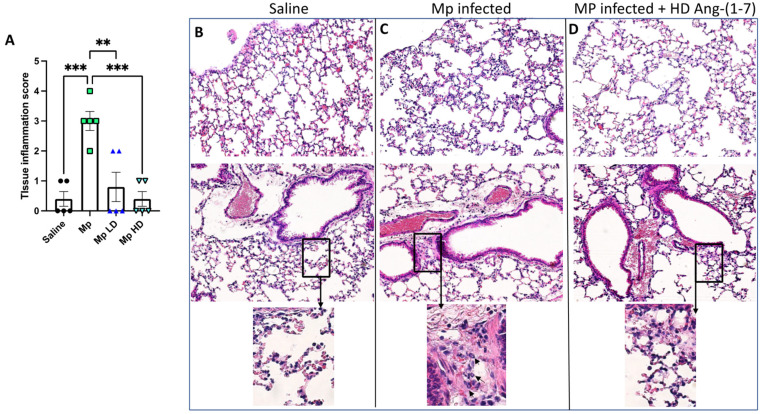
The effect of Ang-(1–7) on pulmonary inflammation in H&E stained slides of sectioned mouse lung. (**A**) Average grade scores for each experimental group. Data are expressed in mean ± SEM. n = 5 mice/group. Representative H&E stained lung sections at 20X magnification from (**B**) saline vehicle, (**C**) Mp infection only (arrows in boxed enlargement indicate neutrophils), (**D**) Mp infection with high dose Ang-(1–7) treatment. ** *p* < 0.01, *** *p* < 0.001 by one-way ANOVA for multiple comparisons.

In order to determine if Ang-(1–7) impacted tissue inflammation during acute Mp infection, left lung sections were processed for H&E staining, and sections of each lung sample were scored according to standard scale. From the four treatment groups described above, sections of each lung sample were scored in a blinded manner according to a standard scale: 0 = only alveolar macrophages were detected throughout entire tissue section; 1 = very few neutrophils observed throughout entire tissue section; 2 = neutrophils present in alveolar airspaces only; 3 = neutrophils observed in alveolar airspaces and in lymphatics; 4 = neutrophils observed in alveolar airspaces, in lymphatics, and in the lumen of large airways. Mice treated with Ang-(1–7) during Mp infection, at both low and high doses, had significantly less lung tissue inflammation compared to Mp-infected mice given vehicle (Figure 4). 

### 3.5. Ang-(1–7) Dose-Dependently Inhibits TNF-α Secretion from RAW 264.7 Cells Following Mp Challenge

**Figure 5:**
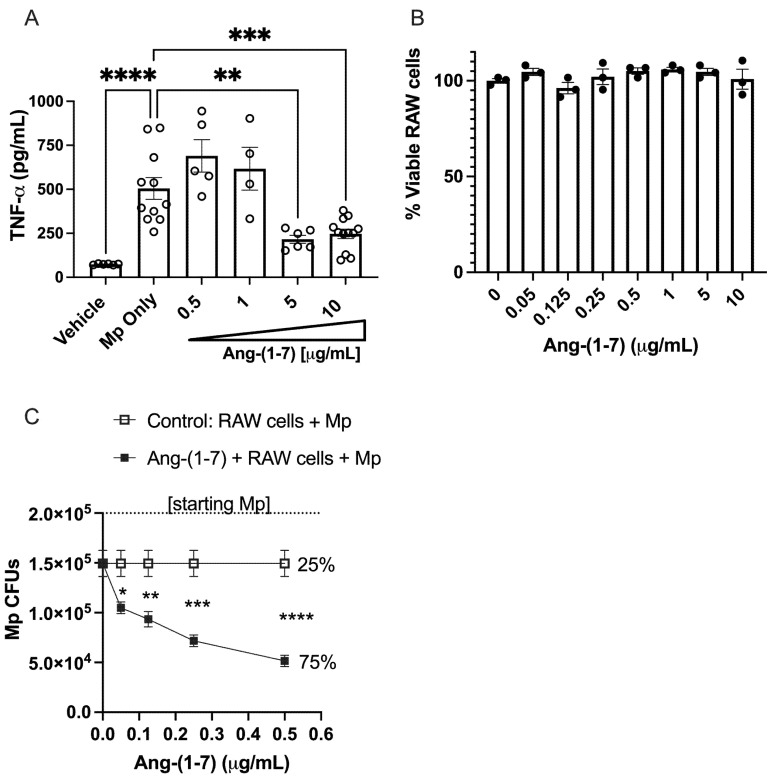
The impact of Ang-(1–7) on macrophage function. (**A**) RAW cells were stimulated with Mp (MOI 10:1) for 4 h in the presence or absence of increasing doses of Ang-(1-7). Cell-free supernatants were assessed for TNF-α by ELISA. n = minimum of two repeats with triplicates for each. (**B**) RAW cells were incubated with increasing doses of Ang-(1–7) in triplicate for 24 h and viability assessed by Trypan blue exclusion using a Countess cell counter. (**C**) RAW cells were stimulated with Mp (MOI of 2:1) with increasing doses of Ang-(1–7) or vehicle control (saline) for 18 h, after which a sample was taken from each well, diluted 1:100 in SP4 media and plated on PPLO agar plates. CFUs were counted after 2 weeks of growth. n = minimum of two repeats with triplicates for each. * *p* < 0.05, ** *p* < 0.01, *** *p* < 0.001, **** *p* < 0.0001 by the *t*-test for each respective dose.

Since macrophages are the predominant producers of TNF-α during airway infection, we sought to determine if Ang-(1–7) would have an impact on Mp-induced TNF-α production in vitro. For these studies, we used a common macrophage cell line, RAW 264.7, which is known to produce high levels of TNF-α. As shown in Figure 5A, Ang-(1–7) had no impact on TNF-α secretion at low doses; however, starting at doses of 5 μg/mL or higher, we did see an inhibition of TNF-α by RAW 264.7 cells. We based our Ang-(1–7) dose response to be within the limits of cell tolerability, as this peptide has not shown toxicity at levels up to 1000 μg/mL in five other cell lines, as recently published [38]. In line with these results, Ang-(1–7) did not reduce RAW 264.7 cell viability at any of the doses tested over a 24 h period (Figure 5B).

### 3.6. Ang-(1–7) Dose-Dependently Promotes Killing of Mp by RAW Cells In Vitro

We next set out to investigate if Ang-(1–7) had an impact on Mp-killing activity in RAW 264.7 cells. In our study, we compared the killing efficiency of RAW cells with and without Ang-(1–7) over an 18 h incubation period. While RAW cells effectively reduced Mp CFUs in media by 25% in 18 h, RAW cells given Ang-(1–7) had a greater reduction in Mp CFUs in a dose-dependent manner, with the lowest dose reducing 48% (* *p* < 0.05 versus RAW cells + vehicle) and the highest dose reducing Mp by 75% (**** *p* < 0.0001 versus RAW cells + vehicle) (Figure 5C). At the dosing range used for these studies, Ang-(1–7) did not appear to directly impact Mp growth alone as no differences in colony counts were detected when Ang-(1–7) was added to Mp alone in the range of 0–0.5 μg/mL (not shown). 

## 4. Discussion

The role of Ang-(1–7) in reducing allergic inflammation in animal models has been previously reported; however, the role of this protective peptide hormone in treating infections common among many chronic asthma sufferers has not been described. *Mycoplasma pneumoniae* is known to colonize the airways of patients considered to have chronic asthma, and this underlying bacterial infection is thought to contribute to asthma exacerbations among these patients. Therefore, we thought it would be important to understand how secondary illnesses common among asthmatics affect the efficacy of a potentially new drug being considered for the treatment of asthma. 

Our research aimed to address how Ang-(1–7) would perform during acute infection with Mp. The main findings of this study indicate that Ang-(1–7) has the ability to provide protection in an experimental mouse model of acute Mp infection by ameliorating inflammatory phenotypes within 24 h of infection. We chose to test Ang-(1–7) delivery to the lungs, as we believed this would have the greatest impact to controlling inflammation. However, additional therapeutic routes, including oral or aerosol delivery via nebulizer, which would be congruent with options for humans, should be explored. In support of this, our colleagues recently reported excellent aerosol dispersion performance of Ang-(1–7) with a human DPI device and in vitro human cell viability assays showed that Ang-(1–7) was biocompatible and safe for different human respiratory cells [38]. We chose to only assess Ang-(1–7) treatment when given shortly (within 2 h) after an infectious challenge. While our findings indicate that high doses of Ang-(1–7) led to decreased infiltrating neutrophils, as well as the cytokines and chemokines that can recruit them, *TNF-α* and *Cxcl1*, we recognize in humans it would be more translatable to give Ang-(1–7) treatment after symptoms of “walking pneumoniae” are more evident, which could occur several days after exposure. Future studies should address the timing for Ang-(1–7) dosing to see how long after Mp infection, delivery of Ang-(1–7) is still efficacious to increase the availability in clinical practices. 

Mp infections among asthmatic patients have been associated with a dramatic spike in levels of TNF-α [39]. We provide evidence that Ang-(1–7) treatment can significantly reduce levels of TNF-α at the RNA and protein levels during Mp infection. In addition, Ang-(1–7) dose-dependently reduced TNF-α production or secretion from Mp-stimulated RAW 264.7 cells, which are a macrophage cell line. While RAW 264.7 cells are well utilized in the field for drug screening, future studies should test Ang-(1–7) on primary macrophage populations from humans to validate our findings. Since macrophages are the predominant producers of TNF-α during respiratory infection, it is relevant that Ang-(1–7) can work directly on these cells during Mp stimulation. Reducing any downstream impacts of TNF-α driven inflammation would likely have additional positive impacts for asthmatic patients beyond those we detected and should be examined in future studies. 

Similarly, mucus production is an important factor when evaluating treatments for asthma, as mucus hyper-secretion into the airway lumen obstructs airways and worsens asthma symptoms [40]. Along these lines, we found that *Muc5AC* gene expression was significantly decreased during Mp infection following treatment with Ang-(1-7). Interestingly, the level of *Muc5AC* was repressed by Ang-(1–7) during infection below the levels normally present without infection. This suggests that Ang-(1–7) may have an inhibitory impact of on mucin production in the absence of infection, which should be further studied. While an increase in mucin production was not evident at this early timepoint, future directions should examine the impact of Ang-(1–7) treatment in longer models of Mp infection to see if the reduced *Muc5AC* gene expression results in deceased mucin production in lung sections. Examination of lung histological sections revealed enhanced neutrophilia in airways of Mp infected mice, while those treated with Ang-(1–7) were more similar to untreated controls. So not only did Ang-(1–7) results in fewer neutrophils migrating into the lung lumen, it also impacted neutrophils migrating into the lung tissue.

Along with providing a promising therapeutic option for the treatment of asthma, the results of this study also indicate that Ang-(1–7) treatment may be helpful for controlling inflammation during Mp infection, and in the clearance of Mp. While fewer neutrophils were detected in the Ang-(1–7) treatment groups, the Mp burden was also decreased. This was somewhat surprising given that neutrophils would contribute to Mp killing in the lungs. This finding of fewer neutrophils and reduced Mp burden, suggest that another compensatory mechanism for pathogen removal may have been activated. Those could include more activation of macrophages prone for pathogen killing or an induction of epithelial-driven antimicrobial host defense. 

We next conducted studies to see if Ang-(1–7) had any direct antimicrobial impact on Mp and we found that Ang-(1–7) alone was not able to reduce Mp CFUs over a two-week growth period (data not shown). However, Mp-killing studies in RAW 264.7 cells in the presence or absence of increasing concentrations of Ang-(1–7) indicated that RAW cells had increased Mp-killing efficiency with increases doses of Ang-(1-7). This suggests that Ang-(1–7) may have a therapeutic potential in stimulating macrophages to better eliminate pathogens in the lungs. Since Ang-(1–7) and analogs are known to reduce ROS and promote NO production, it is likely this Ang-(1–7) activity contributes to the enhanced Mp-killing function by macrophages. Future studies should evaluate this possible mechanism and the ability of Ang-(1–7) to assist in clearance of other infectious agents common among asthmatics, such as human rhinovirus (HRV) and respiratory syncytial virus (RSV) [41]. 

While most commonly known as a vasodilator agent with important roles in the cardiovascular system, Ang-(1–7) has also emerged with anti-oxidant, anti-inflammatory, and anti-fibrotic effects in several model systems [6,42]. Importantly, Ang1-7 inhibits production and expression of many cytokines and adhesion molecules associated with a cytokine storm [43,44] and delivery of exogenous Ang1-7 has been shown to improve oxygenation and can be safely delivered to humans [25,45,46]. In line with these studies, we add that not only does Ang-(1–7) reduce inflammation during live respiratory infection with Mp, it also leads to a significant reduction in Mp pathogen burden and reduction of mucin production in the airways.

## 5. Conclusions

To our knowledge, this is the first study to demonstrate the effects of Ang (1-7) peptide in Mp lung infection and offer mechanistic insight into macrophage killing mechanisms. Taken with the many previous studies in various models in which Ang-(1–7) peptide has anti-inflammatory activity, our studies not only support these findings, but also bring to light a potentially novel indication, i.e., as a therapeutic for the treatment of respiratory infections.

## Data Availability

Not applicable.
